# Effects of glatiramer acetate on fatigue and days of absence from work in first-time treated relapsing-remitting multiple sclerosis

**DOI:** 10.1186/1477-7525-6-67

**Published:** 2008-09-05

**Authors:** Tjalf Ziemssen, Josef Hoffman, Rainer Apfel, Simone Kern

**Affiliations:** 1 MS Center, Neurological University Clinic, Technical University of Dresden, Dresden, Germany; 2 TEVA Germany, Mörfelden, Germany

## Abstract

**Objectives:**

Treatment of multiple sclerosis patients with glatiramer acetate has been demonstrated a beneficial effect on disease activity. The objective of this prospective naturalistic study was to evaluate the impact of glatiramer acetate on fatigue and work absenteeism.

**Methods:**

291 treatment-naïve patients with relapsing remitting multiple sclerosis were included and treated with glatiramer acetate for twelve months. Relapse rates, disability, fatigue symptoms, days of absence from work and adverse events were monitored. Fatigue was measured with the MFIS scale and with a visual analogue scale.

**Results:**

Total MFIS scores decreased by 7.6 ± 16.4 from 34.6 to 27.0 (*p *≤ 0.001). Significant reductions were observed on all three subscales of the MFIS. Fatigue symptoms, assessed using a visual analogue scale, decreased by 1.04 ± 2.88 cm from 4.47 cm to 3.43 cm (*p *≤ 0.001). The proportion of patients absent from work at least once was reduced by a factor of two from 65.1% to 30.1% (*p *≤ 0.001). Tolerance to treatment was rated as very good or good in 78.3% of patients. Adverse effects, most frequently local injection site reactions, were reported in 15.1% of patients.

**Conclusion:**

Treatment with glatiramer acetate was associated with a significant improvement in fatigue symptoms and a marked reduction in absence from work. Treatment was well-tolerated. Such benefits are of relevance to overall patient well-being.

## Introduction

Fatigue is a common symptom of multiple sclerosis [1-5], reported by around three-quarters of affected patients [3], and considered one of the most distressing symptoms of disease by over half [2]. Many patients experience debilitating fatigue every day [2]. In multiple sclerosis, fatigue has a major detrimental impact on quality of life [6-8], is frequently associated with depression [9,10] and is a leading cause of absence from work or impaired work performance [6,11,12]. The pathophysiology of fatigue in multiple sclerosis is poorly understood, but is likely to be multifactorial [13-16]

Treatment of fatigue in multiple sclerosis is thus a major challenge, which cannot be adequately achieved at the present time. Both non-pharmacological and pharmacological interventions have been proposed for the management of fatigue in multiple sclerosis patients [15,17], although the benefits of drugs such as modafenil and amantadine have not been demonstrated unequivocally [18-20].

Immunomodulatory treatments for relapsing-remitting multiple sclerosis, namely glatiramer acetate and the β-interferons, provide a marked reduction in relapse rates and in MRI markers of disease activity [21]. It is therefore of interest to explore whether such treatments might influence fatigue symptoms as well. A retrospective chart review of 218 Canadian patients receiving an immunomodulatory treatment during the late 1990s revealed that fatigue improved over the six months following treatment initiation [22]. Of particular interest was the observation that a significantly higher proportion of glatiramer acetate treated patients than β-interferon-treated patients improved by at least one standard deviation of the Fatigue Impact Scale (FIS).

In order to investigate further the potential impact of immunomodulatory treatment on fatigue in multiple sclerosis, we initiated a prospective, observational, non-interventional study to monitor fatigue in treatment-naive RRMS patients initiating therapy with glatiramer acetate under conditions of daily practice. The primary objective of study was to determine the impact of initiating treatment with glatiramer acetate on fatigue and absenteeism. Secondary objectives were to evaluate the effect of treatment on clinical and MRI outcomes and to determine the tolerability of treatment.

## Methods

This study was a prospective, observational, non-interventional study of patients with relapsing remitting multiple sclerosis treated with glatiramer acetate conducted in Germany. 130 ambulatory and hospital neurologists participated in the study. The study was performed between November 2002 and October 2004.

### Patients

The study included patients with a diagnosis of relapsing-remitting multiple sclerosis by the McDonald criteria [23] who had not previously been treated with an immunomodulatory treatment and in whom the investigator had decided to initiate therapy with glatiramer acetate. Patients were followed for twelve months following treatment initiation.

### Clinical assessment

Patients were evaluated at inclusion and after 3, 6, 9 and 12 months of treatment. At each visit, patients underwent a full neurological assessment, any relapses occurring since the previous visit were ascertained and disability assessed with the Expanded Disability Status Scale (EDSS) [24]. Fatigue was assessed by the patient using a visual analogue scale scored from 0 (no fatigue) to 10 (maximum possible fatigue) and with the Modified Fatigue Impact Scale (MFIS) [25] in its validated German translation. This is a 21-item questionnaire which yields a total score ranging from 0 (no impact of fatigue) to 84 points (maximum impact of fatigue), as well as three subscales representing the physical (score range 0 to 36), cognitive (score range 0 to 40) and psychosocial (score range 0 to 8) dimensions of fatigue.

Patients were questioned about any time spent off work due to their multiple sclerosis. Due to the study protocol, the reasons for work absentism (relapse, fatigue) could not be differentiated. Any adverse events occurring since the previous visit were recorded.

### Statistical analysis

Number of work days lost and fatigue scores over the course of the study were evaluated with the Wilcoxon rank test. All comparisons were two-tailed and a *p *value of < 0.05 was taken as being statistically significant.

### Ethics

This study was conducted according to the Declaration of Helsinki (Hong Kong Amendment) and pertinent national legal and regulatory requirements. Each patient provided written, informed consent and was free to withdraw from the study at any time for any reason without consequences on the care provided.

## Results

### Study sample

A total of 338 patients were included in the study. Of these, 53 were excluded from the analysis due to a protocol violation (24 patients treated previously with an immunomodulatory therapy and 29 patients for whom certain data were recorded retrospectively) and 47 failed to provide complete questionnaire data. The study population thus consisted of 291 subjects (86.1% of included patients).

The baseline demographic and disease variables of the study subjects are presented in Table 1. At inclusion, their median age was 36.9 years and 74.9% were female. The median time since diagnosis was 4.31 years. In the year preceding inclusion, patients had experienced a mean of 1.71 relapses (retrospectively assessed) and their mean EDSS score at inclusion was 2.58. Forty patients (13.7%) discontinued treatment during the course of the study, principally due to the occurrence of an adverse event (sixteen patients).

**Table 1:** Demographic and clinical characteristics of patients at inclusion

	Population (N = 291)
Age (mean ± SD; years)	36.9 ± 9.3

Gender	
Women	218 (74.9%)
Men	67 (23.0%)
*Missing data*	*6 (2.1%)*
	
Time since diagnosis (mean ± SD; years)	4.31 ± 5.47
	
ARR since diagnosis (mean ± SD)	3.82 ± 3.54
No relapses	14 (4.8%)
Up to 2 relapses	111 (38.1%)
3–5 relapses	100 (34.4%)
More than 5 relapses	57 (19.6%)
	
ARR within previous 12 months (mean ± SD)	1.71 ± 0.88
	
EDSS at treatment initiation (mean ± SD)	2.58 ± 1.44
EDSS 0–2	127 (43.6%)
EDSS 3–5	121 (41.6%)
EDSS 6–7	16 (5.5%)
*Missing data*	*27 (9.3%)*

ARR: annualised relapse rate: EDSS: Expanded Disability Status Scale; SD: standard deviation.

### Clinical outcome

Clinical outcome at the end of the study are presented in Table 2. Information on relapses was missing for 24 patients. Of the remaining 267 patients, 61 (22.8%) experienced a single relapse during the twelve-month study period and 23 patients (8.6%) more than one relapse. The mean annual relapse rate during the year of treatment with glatiramer acetate was 0.46. The mean EDSS score at the end of the study was 2.45, representing a mean decrease from baseline of 0.55 points. The change in EDSS score between baseline and twelve months was statistically significant (*p *< 0.05; Wilcoxon rank test). A sustained reduction in EDSS score of > 1 point was observed in fifteen patients (5.2%) and a sustained increase of > 1 point in three patients (1%).

**Table 2:** Clinical outcome

	Population (N = 291)
Relapses during study (*n = 267*)	
No relapse	180 (67.4%)
1 relapse	61 (22.8%)
2 relapses	12 (4.5%)
3 relapses	8 (3.0%)
4–5 relapses	3 (1.1%)
	
Mean EDSS scores (*n = 235*)	
Baseline	2.58 ± 1.45
Study end	2.45 ± 1.52
Change from baseline	-0.13 ± 0.73*

Data are presented as number of patients (%) for relapses and as mean ± SD for Expanded Disability Status Scale (EDSS) scores. The asterisk indicates a significant change from baseline (*p *< 0.05; Wilcoxon signed rank test).

### Fatigue

Overall, 220 patients provided exploitable data from the MFIS questionnaire at both inclusion and study end. Measures were compared between the three-month period before inclusion and the last three months of the treatment period. Significant decreases were observed in the total score as well as in all three dimension scores of the MFIS (Table 3). Similarly, the VAS rating of fatigue was reduced by around one quarter following initiation of treatment with glatiramer acetate (Table 3), between baseline and study end

**Table 3:** Fatigue ratings.

	**Baseline**	**On treatment**	**Mean Change**	*p*
MFIS Total score (n = 220)	34.6 ± 18.7	27.0 ± 18.6	-7.6 ± 16.4	*p *≤ 0.001
Physical dimension score	17.6 ± 9.1	13.5 ± 9.0	-4.1 ± 8.1	*p *≤ 0.001
Cognitive dimension score	13.9 ± 9.2	11.2 ± 8.6	2.7 ± 8.0	*p *≤ 0.001
Psycho-social dimension score	3.1 ± 2.1	2.4 ± 2.0	-0.7 ± 2.0	*p *≤ 0.001

VAS score (n = 198)	4.47 ± 2.53	3.43 ± 2.55	-1.04 ± 2.88	*p *≤ 0.001

Fatigue over three months was measured with the Modified Fatigue Impact Scale (MFIS) and with a visual analogue scale (VAS). Data are presented as mean ± SD for those patients providing exploitable data both at inclusion and at study end. Probabilities were calculated with the Wilcoxon rank test.

### Work absenteeism

The number of days missed from work due to multiple sclerosis was evaluated in the patients who were in employment (72.9% of the study population). In the three month period preceding inclusion, 138 patients (65.1%) had taken at least one day off work (Tables 4 and 5). This number decreased to 64 patients (30.1%) in the year following initiation of treatment with glatiramer acetate. The number of days lost was significantly lower in the second year (*p *≤ 0.001; Wilcoxon rank test).

**Table 4:** Number of days missing from work in the previous year at baseline and one year after start of treatment.

	Baseline		After 12 Months	
	N	%	N	%

No	76	26.1%	148	50.9%
≤ 5 days	26	8.9%	27	9.3%
6–10 days	39	13.4%	14	4.8%
11–20 days	32	11.0%	8	2.8%
> 20 days	50	17.2%	18	6.2%
Not in employment	68	23.4%	62	21.3%
Missing information	0	0%	14	4.8%

**Table 5:** Development of the different groups at baseline (No work absentism, less than 5 days,...) one year after start of treatment with glatiramer acetate using the same categories (No work absentism, less than 5 days,...).

Baseline	No work absentism		≤ 5 days absent		6–10 days absent		11–20 days absent		> 20 days absent		Not in employment	
After 12 Months	N	%	N	%	N	%	N	%	N	%	N	%

No work absentism	59	21.3%	15	5.4%	20	7.2%	23	8.3%	22	7.9%	9	3.3%
≤ 5 days absent	6	2.2%	8	2.9%	8	2.9%	1	0.4%	4	1.4%	0	0%
6–10 days absent	4	1.4%	1	0.4%	3	1.1%	2	0.7%	4	1.4%	0	0%
11–20 days absent	2	0.7%	2	0.7%	1	0.4%	2	0.7%	1	0.4%	0	0%
> 20 days absent	0	0%	0	0%	3	1.1%	1	0.4%	13	4.7%	1	0.4%
Not in employment	3	1.1%	0	0%	2	0.7%	1	0.4%	4	1.4%	52	18.8%

### Safety

Safety was assessed in all 338 included patients. Overall, 51 patients (15.1%) experienced at least one adverse event during the treatment period. These were most frequently injection site reactions or symptoms of a systemic immediate post-injection reaction such as dyspnoea or tachycardia. No single event was reported in more than ten patients. The immediate post-injection reaction was classified as serious in one patient.

## Discussion

In this study, immunomodulatory treatment of relapsing-remitting multiple sclerosis with glatiramer acetate was associated with a reduction in subjective perceptions of fatigue and with the numbers of days taken off work due to illness. We observed a reduction of approximately one-quarter in both MFIS scores and in a VAS measure of fatigue. These findings are consistent with an earlier retrospective study, which also reported an improvement in fatigue measured with the FIS following initiation of glatiramer acetate treatment in 24.8% of patients [22]. The two studies cannot, however, be directly compared due differences in methodology.

The amelioration observed following treatment with glatiramer acetate may be a non-specific consequence of improved overall disease status in treated patients or alternatively result from a specific action of the medication on the pathophysiology of multiple sclerosis fatigue. For example, it has been suggested that fatigue may be aggravated by the production of high levels of pro-inflammatory cytokines [26,27]. The ability of glatiramer acetate to attenuate the secretion and activity of these cytokines within the central nervous system [28,29] may provide such a specific mechanism. Others have proposed, on the basis of magnetic resonance spectroscopy (MRS) findings, that fatigue may be associated with axonal injury in the cortex rather than inflammatory white matter lesions *per se *[30]. In a recent trial, Tedeschi et al. could demonstrate that among MS patients with low disability those with high-fatigue show higher white and gray matter atrophy and higher lesion load. They suggest that in MS, independent of disability, white and gray matter atrophy is a risk factor to have fatigue [31]. In additon, a recent trial of Rocca et al. using functional imaging in MS patients with fatigue and interferon beta-1a treatment pointed out that an abnormal recruitment of the fronto-thalamic circuitry is associated with interferon-induced fatigue in MS patients [32]. In contrast to the interferon's, the specific action of glatiramer acetate to improve MRS markers of axonal injury in multiple sclerosis might contribute to a reduction in fatigue [33,34].

We also observed a dramatic reduction of over fifty percent in the number of patients who needed to take time off work due to their multiple sclerosis. This is consistent with findings from an American study, which also reported a marked decrease in days off work in patients treated with glatiramer acetate [35], but less so with beta-interferons. This is an important functional effect of treatment since the ability to hold down a job satisfactorily is critical for self-esteem and because, in certain countries such as the USA, remaining in full-time employment is an important determinant of obtaining insurance for reimbursement of treatment costs.

Again, the impact of glatiramer acetate on time off work may be an indirect consequence of reduced relapse frequency, although the data from the US study showing a differential effect on time off work between glatiramer acetate and β-interferons would argue against this. Alternatively, the observed effect may be secondary to a reduction in fatigue, which has been identified in other studies to be a major reason why patients with multiple sclerosis need to take time off work [11,12]. Finally, it should be noted that the low incidence of debilitating side-effects reported with glatiramer acetate [36] means that patients are unlikely to need to take time off work due to treatment side-effects.

The strength of this study include the naturalistic design, which means that the findings can probably be generalised to standard care, at least in Europe, with confidence, the prospective nature of the data collection and the relatively large numbers of patients evaluated. Limitations include the absence of a comparator group against which the magnitude of the observed treatment effects could be assessed, and data collection during physician consultations rather than with patients' diaries, which may have introduced some degree of anamnestic error into the findings. The absence of a control group might overestimate the improvement in fatigue symptoms. As a placebo group is probably not ethical it will be further of interest to compare prospectively the benefit on fatigue in a group of naive MS patients treated with GA vs. a group treated with IFN-beta in a next study.

## Conclusion

In conclusion, this non-interventional prospective study demonstrated that treatment with glatiramer acetate was associated with a reduction in patient-reported fatigue ratings and in days missing from work, concomitant with an improvement in clinical manifestations of disease activity. These functional outcomes are of critical importance for overall patient well-being.

## Competing interests

JH and RA are employed by TEVA Germany. TZ has received honoraria and financial compensation by Bayer Healthcare, Biogen Idec, Merck Serono, Pfizer, Sanofi-Aventis and Teva. SK has received honoraria and financial compensation by Bayer Healthcare, Biogen Idec, Sanofi-Aventis and Teva. Research Projects of TZ and SK were funded by the Roland-Ernst-Foundation, Robert-Pfleger-Foundation, Sanofi-Aventis/TEVA and Bayer Healthcare. In the MS center Dresden, clinical studies are performed for Bayer Healthcare, Biogen Idec, BioMS, Genzyme, Glaxo Smith Kline, Sanofi-Aventis and Teva.

## Authors' contributions

RA, JH and TZ were responsible for the conception of the study. TZ drafted the article. All authors contributed to the interpretation of the results and revising the article for important intellectual content. All authors read and approved the final manuscript.
